# ACTIVITIES ACROSS AMERICA: EVALUATING GEOGRAPHIC DIFFERENCES IN ACTIVITY ENGAGEMENT

**DOI:** 10.1093/geroni/igz038.784

**Published:** 2019-11-08

**Authors:** Brittany P Trubenstein, Robin Corley, Kyle D Gebelin, Sergio Rey, Sally Wadsworth, Chandra A Reynolds

**Affiliations:** 1 University of California - Riverside, Riverside, California, United States; 2 University of Colorado – Boulder, Boulder, Colorado, United States

## Abstract

Rurality is associated with cognitive health disparities. We investigated proximal and distal indices of rurality, activity engagement and cognitive performance in the ongoing Colorado Adoption/Twin Study of Lifespan behavioral development and cognitive aging (CATSLife; N = 979; 47% female). The Index of Relative Rurality (IRR) (0 = Urban to 1= Rural) was calculated using population density, population, percent urban, and remoteness at the census tract (IRRtract; M=0.40,SD =.05) and county levels (IRRcounty; M=0.53, SD=.09), which were moderately correlated (r = .21, p = .000). Individuals reported weekly-hours of engagement in 19 activities, classified into social (M=6.85, SD=4.03), physical (M=6.53, SD=4.76), family (M=10.76, SD=7.06), sedentary (M=11.84, SD=5.83), or cognitive (M=4.63, SD=3.74) domains. Social activities correlated with IRRcounty (r=0.091, p = .005) but not with IRRtract (r=-0.004). WAIS-III IQ scores were available. Social activities modestly correlated with IQ, particularly Verbal-IQ (r = .063, p = .049). Cognitive activities correlated with all IQ measures (r’s = .17 to .25, p < .000). While IRRcounty correlated positively with IQ (r’s=0.057 to 0.094, p’s = .079 to .000), IRRtract correlated negatively but not significantly with IQ (r’s=-0.053 to -0.062, p’s = .104 - .054). Analyses accounting for family nesting, sex, and age suggested compensatory associations between IRRcounty versus IRRtract and Full-Scale-IQ (p < .019), with similar patterns for Verbal-IQ and Performance-IQ. Social activities did not uniquely contribute. Further investigation is warranted to better understand the complex relationships between proximal and distal rurality and the implications that these relationships have on activity engagement and cognitive performance.

